# Digital solutions for assisting cancer patients manage the physical, emotional, psychological and social complications

**DOI:** 10.15190/d.2019.8

**Published:** 2019-06-30

**Authors:** Chad Walkaden

**Affiliations:** Counselling and Consulting Services, New South Wales, 2099, Australia

**Keywords:** Digital solution, cancer blueprint, management of complications in cancer patients.

## Abstract

The introduction of immunotherapy as a treatment option has been a significant contributor to improving the survival rates for certain cancer patients. Notwithstanding these astonishing achievements, there are novel challenges for overstretched healthcare systems that will be required to manage the complex medical needs of a projected 34% increase in the number of cancer survivors over the next seven years. These alarming figures highlight the need for health systems to strengthen their capacity to deliver effective digital solutions that can be scaled to support to patients with their range of medical needs. At the core of the provision of digital solutions, it appears that a need exists for a dual focus to exist whereby patients and health care system equally benefit from the introduction of services. Among the available initiatives is a cancer support program, “The Cancer Blueprint”. The Cancer Blueprint has passed three stages of testing and has impressive results that shows significant potential to be a major part in the future of cancer support programs.

In the USA alone, the annual number of people diagnosed with cancer is an estimated 1,735,350^1^. While this number is alarming, the continual advancements in oncology treatment options are increasingly improving the survival rates for some of these patients. The introduction of immunotherapy as a treatment option has been a significant contributor to these changing trends. In some cases, dramatic results have been observed for patients being treated with immunotherapies, indicating that it is feasible to restore effective antitumor immune surveillance^2^. More remarkably, for specific cancers types, immunotherapy has been able to quadruple 5-year survival of certain patients compared to standard chemotherapy^3^. 

Notwithstanding these astonishing achievements, there are novel challenges for overstretched healthcare systems that will be required to manage the complex medical needs of a projected 34% increase in the number of cancer survivors over the next seven years^3^. Additionally, according to emerging scientific, health systems will not merely be impacted by the increase in volume of patients, but health systems will also need to manage the care needs of cancer survivors that are known to have an increased risk of comorbidity from diseases including diabetes, cardiovascular disease and mental illnesses that include but are not limited to depression and anxiety disorders^4^. These alarming figures highlight the need for health systems to strengthen their capacity to deliver effective digital solutions that can be scaled to support to patients with their range of medical needs. 

Recently, hospitals, pharmaceutical companies, health systems, charities, and a range of startups have attempted to play a key role in integrating digital solutions into the current landscape for patients. While there is no recognized market leader, the increasing availability of options signals that there will be a continual push to incorporate digital solutions into the care and management of cancer patients and cancer survivors. Among the initiatives includes CancerAid, the startup that has digitalized the access of patient records, “Stupid Cancer” an application to provide practical support for patients and “The Cancer Blueprint”, a cancer support program that has passed three stages of early testing to show a capacity to reduce isolation, reduce anxiety and also improve the capacity of patients to cope. 

At the core of the provision of digital solutions, it appears that a need exists for a dual focus to exist whereby patients and health care system equally benefit from the introduction of services. For patients, there is a requirement that they can access support that is effective at assisting them with practical support as well as the accessibility of personalized mental health support, symptom management and the provision of evidence-based integrative strategies to assist in the improvement of short-term care management and the prevention of the risks of developing secondary illnesses. For healthcare systems, a primary factor driving the introduction of digital solutions is the need to have more cost-effective ways that they can meet the expected rising costs associated with the increase in volume of patients and cancer survivors. For this to occur, more rigorous testing of these digital solutions are required to measure the possible financial savings while at the same time demonstrating the capacity of these solutions to improve key markers that includes increasing patient quality of life and increasing their overall survival. 

The Cancer Blueprint has been one initiative attempting to support the availability of data. In the most recent evaluation, 100 participants were introduced to a digital version of this cancer support program. In the pilot, participants received specific content to target their physical, emotional and psychological well-being in bite-sized information directly to their phone and or computer. The participants were scored across key domains that included: (1) the impact of cancer on their life, (2) optimism, (3) quality of life, (4) worry about the future, (5) isolation, (6) capacity to cope. While further study is required on this initiative and the many other cancer support options, the following conclusions were drawn: (a) from completing the pilot, patients reported that their anxiety and isolation almost halved and that their capacity to cope significantly improved; (b) for the three remaining key measures, the patients measured the same or had slight improvements; (c) there are significant indicators that show the potential for The Cancer Blueprint to play a major part in the future of cancer support programs. 

These impressive results were obtained solely through the content without additional features or technological integration with other devices that would likely strengthen the capacity of the program to better support patients and be more appealing to healthcare systems interested in delivering scalable cost saving solutions. 
